# Trophic ecology of sea urchins in coral-rocky reef systems, Ecuador

**DOI:** 10.7717/peerj.1578

**Published:** 2016-01-14

**Authors:** Nancy Cabanillas-Terán, Peggy Loor-Andrade, Ruber Rodríguez-Barreras, Jorge Cortés

**Affiliations:** 1 Departamento Central de Investigación, Universidad Laica Eloy Alfaro de Manabí, Ciudadela Universitaria. Vía San Mateo, Manta, Manabí, Ecuador; 3 Department of Biology, University of Puerto Rico at Bayamón, Puerto Rico; 4 Centro de Investigación en Ciencias del Mar y Limnología (CIMAR), and Escuela de Biología, Universidad de Costa Rica, San Pedro, San José, Costa Rica

**Keywords:** *Diadema mexicanum*, *Eucidaris thouarsii*, Stable isotopes, Rocky reefs, Niche breadth, Eastern Tropical Pacific

## Abstract

Sea urchins are important grazers and influence reef development in the Eastern Tropical Pacific (ETP). *Diadema mexicanum* and *Eucidaris thouarsii* are the most important sea urchins on the Ecuadorian coastal reefs. This study provided a trophic scenario for these two species of echinoids in the coral-rocky reef bottoms of the Ecuadorian coast, using stable isotopes. We evaluated the relative proportion of algal resources assimilated, and trophic niche of the two sea urchins in the most southern coral-rocky reefs of the ETP in two sites with different disturbance level. Bayesian models were used to estimate the contribution of algal sources, niche breadth, and trophic overlap between the two species. The sea urchins behaved as opportunistic feeders, although they showed differential resource assimilation. *Eucidaris thouarsii* is the dominant species in disturbed environments; likewise, their niche amplitude was broader than that of *D. mexicanum* when conditions were not optimal. However, there was no niche overlap between the species. The Stable Isotope Analysis in R (SIAR) indicated that both sea urchins shared limiting resources in the disturbed area, mainly *Dictyota* spp. (contributions of up to 85% for *D. mexicanum* and up to 75% for *E. thouarsii*). The Stable Isotope Bayesian Ellipses in R (SIBER) analysis results indicated less interspecific competition in the undisturbed site. Our results suggested a trophic niche partitioning between sympatric sea urchin species in coastal areas of the ETP, but the limitation of resources could lead to trophic overlap and stronger habitat degradation.

## Introduction

As a consequence of increasing human pressure, coastal ecosystems are facing a wide range of threats, such as resource exploitation and habitat modification (Wilkinson, 1999; Dumas et al., 2007; Costello et al., 2010; Rossi, 2013). Several studies have evaluated the development of rocky bottom disturbances by analyzing the densities of echinoids and the development stage of habitats (Phillips & Shima, 2006; Alvarado, Cortés & Reyes-Bonilla, 2012; Hereu et al., 2012). Some of these studies have correlated different phases of benthic substrate degradation, considering sea urchin density and their association with functional algae groups (Steneck, 1983; Steneck & Dethier, 1994). Another approach to decipher benthic dynamics is through the trophic relationships between consumers and resources using stable isotopes (Behmer & Joern, 2008). Stable isotope analysis (SIA) has been a powerful tool to study trophic ecology, especially for those species with foraging habits for which it is difficult to use traditional techniques, such as stomach content analyses. Several studies have focused on sea urchins from a stable isotope approach (e.g., Minagawa & Wada, 1984; Tomas et al., 2006; Vanderklift, Kendrick & Smith, 2006; Wing et al., 2008; Cabanillas-Terán, 2009; Rodríguez-Barreras et al., 2015).

Stable carbon and nitrogen isotope ratios provide time-integrated information regarding feeding relationships and energy flow through food webs (DeNiro & Epstein, 1981; Peterson & Fry, 1987; Vander-Zanden & Rasmussen, 2001; Carabel et al., 2006). Stable isotopes can be used to study the trophic niche of a species due to the “*δ*-space.” This is comparable to the *n*-dimensional space that ecologists refer to as a niche because an animal’s chemical composition is directly influenced by what it consumes, as well as the habitat in which it lives (Newsome et al., 2007; Parnell et al., 2010; Boecklen et al., 2011).

Carbon is a conservative tracer used to track energy sources in food webs, while nitrogen helps determine the trophic position (Minagawa & Wada, 1984; Vander-Zanden & Rasmussen, 2001; Post, 2002; Phillips, 2012; Phillips et al., 2014). Carbon (*δ*^13^C) and nitrogen (*δ*^15^N) stable isotopes have been used in marine ecosystems to determine the food habits of species (Peterson & Fry, 1987), nutrient migrations within food webs, the trophic position of organisms and their contribution at every level (Vander-Zanden & Rasmussen, 1996), the origin and transformation of the ingested organic matter (Peterson, Howarth & Garrett, 1985), or how some ecosystems have organisms that occupy similar trophic positions coexisting in high densities (Vanderklift, Kendrick & Smith, 2006). Moreover, SIA are useful to assess the ecosystem health (e.g., Cole et al., 2004; Hamaoka et al., 2010; Karube et al., 2010). For example, human influence on lake ecosystems were studied by Karube et al. (2010) and those authors found that signatures of *δ*^13^C and *δ*^15^N in macroinvertebrates of the littoral zone are indicators of anthropogenic impacts from the watershed. Inorganic nitrogen loading from the watershed was recorded in *δ*^15^N of snails.

Reef degradation currently has significant consequences for morpho-functionality of marine environments (Hoegh-Guldberg, 1999; Mumby, Foster & Fahy, 2005), and the Ecuadorian reefs are no exception (Glynn & Wellington, 1983; Glynn, 1993; Guzmán & Cortés, 1993; Glynn, 2003). Anthropogenic stressors can have synergistic effects on reefs, such as the harmful algae blooms that are becoming increasingly important drivers of variation in the sea urchin populations, as seen in other areas (Hunter & Price, 1992; Lapointe et al., 2005; Lapointe et al., 2010).

Coral-rocky reefs have rarely been studied along the Ecuadorian mainland, despite the serious threat by eutrophication, fisheries and other anthropogenic impacts (Guzmán & Cortés, 1993; Cortés, 1997; Cortés, 2011). Ecuadorian coral communities are important because they represent the southernmost distribution in the Eastern Tropical Pacific (ETP). Ecuador has no extensive reef systems, as the majority of reefs are small rocky patches with some coral colonies. Nevertheless, those areas are characterized by high biodiversity, including more than a quarter of the Ecuadorian continental fishes and a great number of echinoderms, sea fans, and scleractinian corals (Glynn et al., 2001; Glynn, 2003; Rivera & Martínez, 2011).

Sea urchins have the capability to modify the community structure through foraging, as several authors have previously mentioned (e.g., Carpenter, 1981; Carpenter, 1986; Hay, 1984; Hay & Fenical, 1988; Sala, Boudouresque & Harmelin-Vivien, 1998), and we need to elucidate what occurs in areas where there are more than one sea urchin species which dominate the substratum and their role in controlling fleshy macroalgae. The sea urchins *Diadema mexicanum* (Agassiz, 1863) and *Eucidaris thouarsii* (Agassiz & Desor, 1846) are two of the most dominant benthic grazers in the ETP (Guzmán & Cortés, 1993). These two echinoids exert a strong influence on the community structure (Lawrence, 1975; Glynn, Wellington & Birkeland, 1979; Andrew, 1989; Underwood, 1992). In the ETP, the sea urchin *E. thouarsii* could be described as a major herbivore in rocky reef bottoms. Its preferential resource appeared to be benthic algal turf and macroalgae, but if those were not available, it feeds on other organisms, such as the corals *Pavona clavus*, *Pocillopora* spp. and *Porites lobata* (Glynn, Wellington & Birkeland, 1979; Glynn & Wellington, 1983; Reaka-Kudla, Feingold & Glynn, 1996).

The ratios of *δ*^15^N and *δ*^13^C in consumers are strongly influenced by their food resources (Phillips et al., 2014) and it is necessary to identify their ecological role, not only by their capacity to structure the environment, but to understand the dynamics of coexistence of the sea urchin populations along the Ecuadorian coast. The relative position of *δ*^13^C vs. *δ*^15^N echinoids species can be displayed in a bi-plot and help to understand food web structure and organism responses to niche shifts, diet variability and human impact (Layman et al., 2007). The aim of this study was to improve the knowledge and understanding of the trophic biology of *D. mexicanum* and *E. thouarsii* in Ecuadorian rocky reefs. We determined the stable isotopes of carbon and nitrogen isotope for both sea urchin species. The complexity of the littoral zone was analyzed using stable isotopes to understand the trophic interactions of these two echinoids in areas with different degree of human impact. We assume that the more developed rocky-reef and substrate associated to coral coverage will favor habitats with complex trophic interactions (see Duffy et al., 2007; Alvarez-Filip et al., 2009; Graham & Nash, 2013), resulting in wider isotopic echinoids niche breadth.

## Material & Methods

### Site descriptions

**Figure 1:**
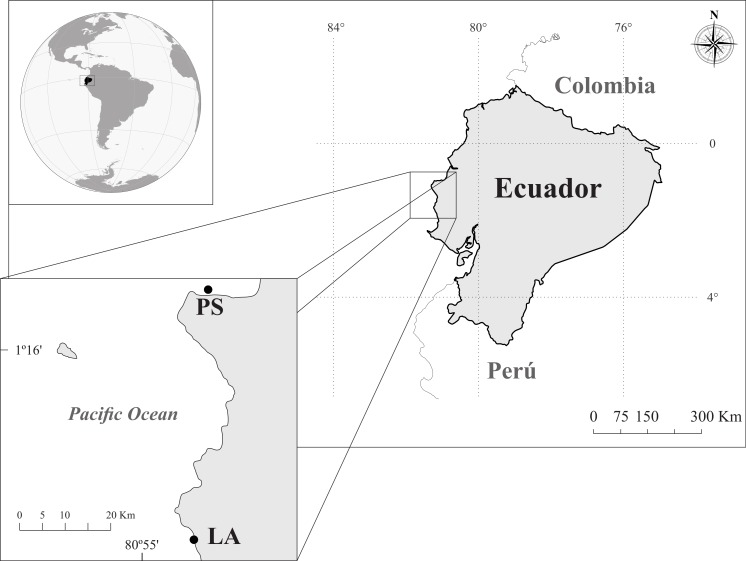
Study area and sampling sites in the coast of Ecuador: Los Ahorcados (LA) and Perpetuo Socorro (PS).

This study was conducted between May to September of 2013, at two localities, Los Ahorcados (LA: 01°40′42″S; 80°51′58″W) and Perpetuo Socorro (PS: 0°55′40″S; 80°44′25″W), in Manabí province, Ecuador (Fig. 1). The LA site was a small group of rocky islets located near Machalilla National Park. Although this area was not considered a protected area, it had a very high diversity of scleractinians and octocorals (Rivera & Martínez, 2011). LA presented a rocky bottom with clear geomorphologic differences between the leeward and windward areas. The leeward area were 25 m depth, and the windward side was mainly build by octocorals (22 species), and hexacorals, such as *Pavona* spp., the branching corals *Pocillopora* spp. and solitary corals. The PS site is located in front of the Port of Manta (1.5 km), one of the most important ports in Ecuador for large pelagic fisheries (Villón & Beltrán, 1998a; Villón & Beltrán, 1998b; Martínez-Ortiz et al., 2007) and greatly impacted by anthropogenic activities (see details in Table 1). PS rocky reef is a homogenous bottom of 7–9 m depth, and had a substrate consisted mainly of a mixture of rock and sand with scarce scleractinian corals (*Pavona* spp. and *Pocillopora* spp.) and gorgonians (mainly *Leptogorgia alba*).

In order to distinguish both sites, a coral-rocky reef category was used for this study, which was developed taking into account habitat complexity and type of disturbance to establish two categories, namely disturbed and undisturbed (Table 1). The distance of human impact to the sites, size of the fleet and type of human disturbance were considered. The rugosity index (RI), which is the ratio of a length of chain following the reef contour to the linear distance between its start and end point (modified of Risk, 1972) was used. To calculate the RI, we used a three-meter chain five times equitably-distributed along 15 transects (20 m). The average RI obtained with transects was used to determine the rugosity level per site, where larger numbers indicate higher complexity following Alvarez-Filip et al. (2009) and Alvarez-Filip et al. (2011). Therefore, values of *RI* < 1.5 were considered low complexity and *RI* > 1.5 were defined as complex.

**Table 1:** Category of coral-rocky reef sites and source of human impact.

Site	Source of human impact	Human population density (ind km^−2^)	Distance from sampling site to Source of human impact (km)	Rugosity index (RI)	Category
Los Ahorcados (LA)	Artesanal fishery + hotel zone	54.55	17.24	2.32	Undisturbed
Perpetuo Socorro (PS)	Artesanal and industrial Fishery + hotel zone + industry discharge	1046.34	3.43	1.10	Disturbed

### Collecting and processing data

We collected algal samples for identification, to calculate biomass, and to carry out SIA. Algal biomass was measured using twelve 50 × 50 cm quadrats per site. The quadrats were located randomly within the sea urchin habitat. The substrate inside each quadrat was scrapped, carefully removed, collected in bags, and frozen for later analysis. Macroalgae were identified to the lowest possible taxonomic level using the available keys (Abbott & Hollenberg, 1976; Afonso-Carrillo & Sansón, 1999; Littler & Littler, 2010). The sampled invertebrate and algal species for this study are not threatened. The necessary permits were obtained from the Ministry of Environment of Ecuador (014AT-DPAM-MAE).

In areas where the algal cover was dominated mainly by turf species (following the morpho-functional category of Guidetti, 2006), we used a sniffer with a dense mesh bag coupled to a compressed air tank. In the laboratory, individuals were separated into species and gently washed with distilled water and dried in an oven at 50 °C for 24 h to measure the dry weight.

We collected four individuals of *D. mexicanum* and six of *E. thouarsii* in LA and twelve individuals of *D. mexicanum* and eight of *E. thouarsii* in PS at the same depth range (8–10 m). Only individuals greater than 5.0 cm in test diameter were collected to avoid any effect of the development stage. The samples were frozen shortly after collection and processed at the laboratory. The muscles of Aristotle’s lanterns were removed carefully and washed from the stomach contents to estimate algal assimilation by *D. mexicanum* and *E. thouarsii*. This tissue provides a time-integrated measure of assimilated sources (e.g., Michener & Schell, 1994; Ben-David & Schell, 2001; Polunin et al., 2001; Phillips & Koch, 2002; Rodríguez, 2003; Tomas et al., 2006).

The algal and echinoids muscle samples were rinsed with filtered water, dried at 50 °C during 36 h, ground to a fine powder and placed in glass vial for isotope analyses. To remove carbonates from some algal species (*Lobophora variegata* and *Polysiphonia* spp.), the samples were washed with diluted HCl at 1 N prior to drying to avoid disturbance in the mass spectrometer reading. A subsample was taken of each alga and muscle (∾1 mg) to evaluate the ^13^C/^12^C and ^15^N∕^14^N ratios using a Thermo Electron Delta V Advantage Mass Spectrometer. Carbon and nitrogen samples were analyzed in a dual isotope mode at the Geology Department, University of Florida, Gainesville, Florida.

The isotope samples were loaded into Eppendorf capsules and placed in a 50-position automated Zero Blank sample carousel on a Carlo Erba NA1500 CNS elemental analyzer. After combustion in a quartz column at 1,020 °C in an oxygen-rich atmosphere, the sample gas was transported in a He carrier stream and passed through a hot reduction column (650 °C) consisting of elemental copper to remove oxygen. The effluent stream then passed through a chemical (magnesium perchlorate) trap to remove water, followed by a 3 m Gas chromatography (GC) column at 45 °C to separate N_2_ from CO_2_. The sample gas next passed into a ConFlo II preparation system and into the inlet of a mass spectrometer running in continuous flow mode, where the sample gas was measured relative to laboratory reference N_2_ and CO_2_ gases. The carbon isotopic results were expressed in standard delta notation relative to Vienna Pee Dee Belemnite (VPDB). The nitrogen isotopic results were expressed in standard delta notation relative to atmospheric air. The standard deviations of *δ*^13^C and *δ*^15^N replicate analyses were estimated; the precision values were 0.074 and 0.148 for carbon and nitrogen isotope measurements, respectively. Carbon and nitrogen samples were analyzed in a dual isotope mode. Ratios are expressed as:

\begin{eqnarray*} \delta X(\text{ \permil})=[({R}_{\mathrm{sample}}{}{R}_{\mathrm{standard}})-1]\times 1,0 0 0;\text{where}\hspace{1em}X={}^{1 3}\mathrm{C}~\text{or}{~}^{1 5}\mathrm{N}~\text{and}~{R}_{\mathrm{ sample}}={}^{1 3}\mathrm{C}{{}}^{1 2}\mathrm{C}\hspace{1em}{\text{or}\hspace{1em}}^{1 5}\mathrm{N}{{}}^{1 4}\mathrm{N}. \end{eqnarray*}

### Data analysis

**Figure 2:**
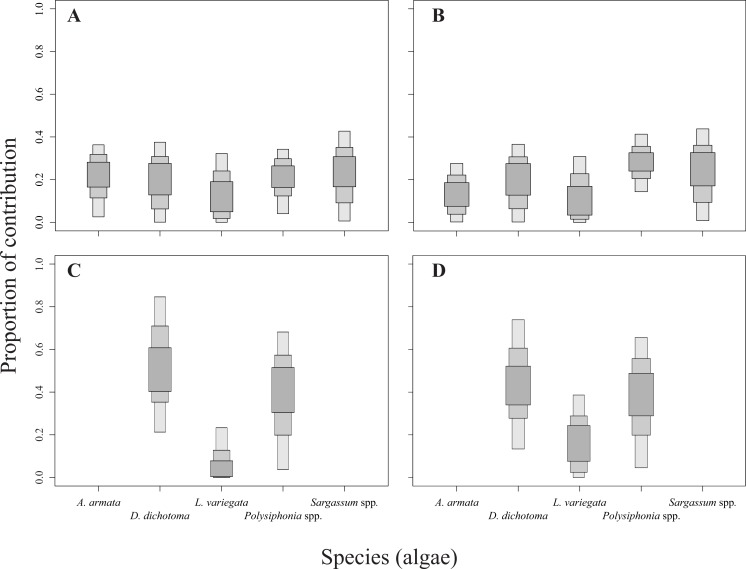
Contribution rates of algae to the diet of the two sea urchin species. Results are shown as 25, 75 and 95% of credibility intervals. (A) Represents the contribution for *Diadema mexicanum* in Los Ahorcados (LA), (B) for *Eucidaris thouarsii* in LA, (C) *D. mexicanum*in Perpetuo Socorro (PS), and (D) *E. thouarsii* in PS.

The relative contribution of algae to the diet of the sea urchins *D. mexicanum* and *E. thouarsii* was estimated with a Bayesian isotopic mixing model (SIAR, Parnell & Jackson, 2013), which included the isotopic signatures, fractionation and variability to estimate the probability distribution of the contribution of the food source to a mixture. This procedure supplied accurate information about the contribution of algal species to the sea urchin tissues recognized the main components of the diet under different conditions (Peterson, 1999; Fry, 2006; Wing et al., 2008). Lipid extraction in sea urchins was not necessary since Aristotle lantern’s muscle is low in lipids, on the other hand when the C:N ratios are lower than 3.5 it is not recommended (Post et al., 2007) see Table S1. The isotopic discrimination factor values used to run the model were 2.4 ± 1.6‰ (mean ± SD) for *δ*^15^N, and 0.4 ± 1.3‰ for *δ*^13^C (Fry & Sherr, 1984; Minagawa & Wada, 1984; Michener & Schell, 1994; Moore & Semmens, 2008). The results of the mixing model showing the calculated sea urchin dietary proportions were represented as box plots, indicating the 25%, 75%, and 95% of credibility intervals (Fig. 2).

The niche width and overlap for the sea urchins were estimated with Stable Isotope Bayesian Ellipses in R (SIBER) (Jackson et al., 2011) from the SIAR package (Parnell & Jackson, 2013). This analysis uses metrics based on ellipses and provides the standard ellipse corrected area (SEA_c_) used as the trophic niche breadth and the overlap between ellipses, where values close to 1 represent a higher trophic overlap.

Prior to the statistical analysis, the homogeneity and normality of variance were tested by performing a Kolmogorov–Smirnov and a Cochran’s test (Zar, 2010). Statistical difference was performed comparing *δ*^15^N and *δ*^13^C values between species. In addition, the algal biomass between sites was evaluated with a one-way ANOVA, with site as a fixed factor. The statistical analyses were performed using R with an alpha of 0.05 (R Core Team, 2014).

## Results

The benthic communities in Ecuadorian rocky reefs ranged between habitats dominated by macroalgae and live corals (LA), and habitats dominated by turf and coral skeletons (PS). Site estimates for perturbation and complexity are outlined in Table 1. LA is a site with structural complexity and dominance of branched erect algae, while PS has low structural complexity and dominance of turf (Table 1).

The algae collected in LA were *Asparagopsis armata*, *Dictyota dichotoma*, *Lobophora variegata*, *Polysiphonia* spp., and *Sargassum* spp., while in PS were *D. dichotoma, L. variegata*, and *Polysiphonia* spp. The greatest algal biomass was recorded for *L. variegata* at both sites, while *D. dichotoma* was the algae with the lowest biomass at both localities (Table 2). Overall, the biomass average values ranged from 35.8 ± 9.73 g (dry weight) m^−2^ for PS to 143.00 ± 20.67 g m^−2^ in LA. We found significant differences between both sites (ANOVA, df = 1, *F* = 3.60, *p* < 0.01). The overall algal *δ*^15^N fluctuated from 5.05 to 9.49‰ (Table 3). PS displayed the highest mean values of nitrogen with *D*. *dichotoma* (7.60 ± 0.53‰). At LA, *Polysiphonia* spp. exhibited the highest mean value for nitrogen (7.19 ± 1.13‰). We found significant differences in *δ*^15^N between sites (ANOVA, df = 1, *F* = 5.29, *p* = 0.02), taking into account all the algae isotopic signatures. As for *δ*^13^C, ratios fluctuated from −23.65 to −6.90‰, with LA displaying the most negative values (*A. armata*). There was no significant difference in *δ*^13^C among sites (ANOVA, DF = 1, *F* = 1.41, *p* > 0.05).

**Table 2:** Average algal biomass in grams (dry weight) m^−2^ ± standard deviation at Los Ahorcados (LA) and Perpetuo Socorro (PS)

Species	LA	PS
*A. armata*	34.32 ± 16.98	–
*D. dichotoma*	4.69 ± 1.90	0.60 ± 0.20
*L. variegata*	66.77 ± 24.52	23.26 ± 12.61
*Polysiphonia* spp.	30.73 ± 12.82	16.38 ± 6.26
*Sargassum* spp.	5.94 ± 3.09	–

**Table 3:** Mean ± standard deviation values of *δ*^13^C and *δ*^15^N of algal genus considered in the mixing model analysis taken from Los Ahorcados and Perpetuo Socorro.

	Los Ahorcados			Perpetuo Socorro	
Species	*δ*^13^C	*δ*^15^N	**Species**	*δ*^13^C	*δ*^15^N
*A. armata* (*n* = 4)	−23.63 ± 0.10	5.68 ± 0.02	–	–	–
*D. dichotomaa* (*n* = 4)	−17.30 ± 1.94	6.65 ± 0.791	*D. dichotoma* (*n* = 3)	−15.27 ± 3.05	7.60 ± 0.53
*L. variegata* (*n* = 4)	−15.73 ± 3.331	5.89 ± 0.638	*L. variegata* (*n* = 3)	−12.02 ± 0.60	7.06 ± 1.08
*Polysiphonia* spp. (*n* = 6)	−9.33 ± 1.759	7.19 ± 1.129	*Polysiphonia* spp. (*n* = 4)	−14.72 ± 3.04	7.38 ± 0.36
*Sargassum* spp. (*n* = 4)	−18.30 ± 0.07	6.97 ± 0.06	–	–

Values of *δ*^15^N were particularly different between the two species of sea urchins (ANOVA, df = 1, *F* = 20.10, *p* < 0.001). The isotopic value of *δ*^15^N for *D. mexicanum* ranged from 11.38 to 12.99‰, whereas *E. thouarsii* displayed values from 12.31 to 14.15‰. The average values of *δ*^13^C and *δ*^15^N estimated for *D. mexicanum* in LA were −16.67 ± 0.04 and 11.53 ± 0.14‰, respectively, while *E. thouarsii* displayed −15.46 ± 0.16‰ and 12.84 ± 0.40‰, respectively. In PS, the *D. mexicanum* isotopic signals were −16.25 ± 0.39‰ for *δ*^13^C and 12.62 ± 0.22‰ for *δ*^15^N; while *E. thouarsii* displayed −15.41 ± 0.43‰ for *δ*^13^C and 13.54 ± 0.47‰ for *δ*^15^N. We found significant differences in *δ*^13^C between species (ANOVA, df 1, *F* = 49.31, *p* < 0.0001), and the most negative values were found at LA. The *δ*^15^N showed the same patterns as those algae (higher values for PS). *δ*^15^N ratios of both sea urchins differed between the study sites, as LA reported lower values than PS (ANOVA, df 1, *F* = 7.59, *p* < 0.01). The most notorious difference was due to *D. mexicanum* (ANOVA, df 1, *F* = 82.41, *p* < 0.0001).

**Figure 3:**
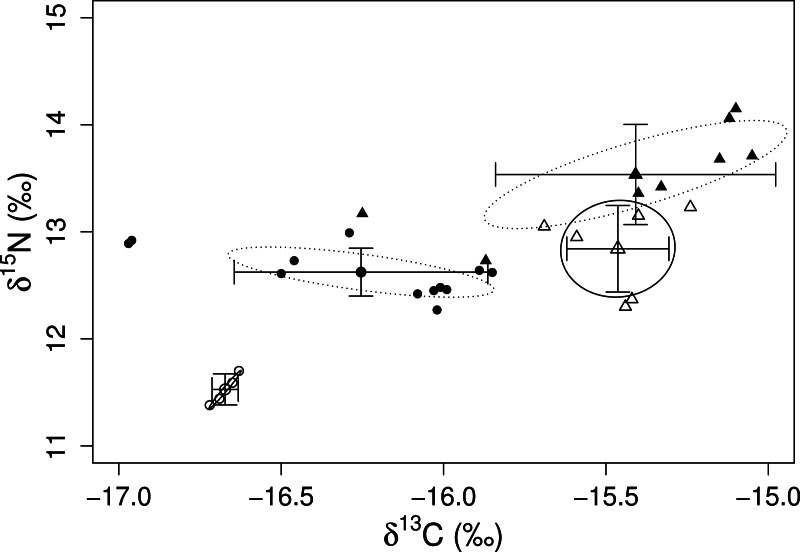
Isotope niche breadth of the echinoids, *D. mexicanum* (circles) and *E. thouarsii* (triangles) in Los Ahorcados (white symbols and solid line) and Perpetuo Socorro (black symbols and dotted line).

The mixing models provided evidence for the contribution of different algal resources for both sites and species. The SIAR analysis showed that *Sargassum* spp. was the most important resource for *D. mexicanum* in LA (up to 43%), followed by *D. dichotoma* and *A. armata* as secondary resources (up to 37% for both). Likewise, *Sargassum* spp. was the main algal resource for *E. thouarsii* in the same locality (up to 44%), followed by *Polysiphonia* spp. (up to 41%) (Table 4). Contrasting, at PS the main macroalgal contributor was *D*. *dichotoma* for both sea urchins (Fig. 2), with up to 85% of the proportional contribution for *D. mexicanum* and close to 75% for *E. thouarsii.*Table 5 shows data on isotopic niche breadth as measured by the corrected standard ellipse area (SEAc). The main difference in the trophic niche breadth was caused by *E. thouarsii* with a difference probability of 52%; overlap between species isotopic niches was not found in any case (Fig. 3), but the SEAc was higher for *E. thouarsii* in both sites with 0.25 in LA and 0.46 in PS (Table 5).

**Table 4:** Average percentage (%) contribution of algal **species** to the diet of the sea urchins *D. mexicanum* and *E. thouarsii* at Los Ahorcados (LA) and Perpetuo Socorro (PS) produced by the SIAR model using isotope values from algae. Minimum and maximum values for each algae are shown in parentheses.

	*Diadema mexicanum*		*Eucidaris thouarsii*	
Species	LA	PS	LA	PS
*A. armata*	21 (2–37)	–	14 (0–28)	–
*D. dichotoma*	20 (0–37)	52 (21–85)	19 (0–37)	44 (13–75)
*L. variegata*	16 (0–32)	9 (0–23)	15 (0–31)	19 (0–38)
*Polysiphonia* spp.	20 (04–35)	38 (3–67)	28 (15–41)	38 (4–66)
*Sargassum* spp.	23 (1–43)	–	24 (1–44)	–

**Table 5:** Trophic niche breadth of sea urchins calculated by SIBER analysis of muscle values. SEAc: corrected standard ellipse area. The right column shows statistical differences in SEA.

Species	SEAc	Ellipses areas: group differences probability (%)
*D. mexicanum* (LA)	0.005	1 vs. 2 (10.4)^a^
*D. mexicanum* (PS)	0.218	
*E. thouarsii* (LA)	0.250	1 vs. 2 (52.0)^a^
*E. thouarsii* (PS)	0.457	

**Notes.**

a Group 1: Los Ahorcados (LA); Group 2: Perpetuo Socorro (PS).

## Discussion

There is very little information on the ecology of the Ecuadorian coast, and no data pertaining to trophic relationships among sea urchins, apart from this study. The majority of the available information on Ecuador came from studies conducted on Galapagos reefs (Glynn, Wellington & Birkeland, 1979; Glynn & Wellington, 1983; Glynn, 2003; Glynn, 2004; Glynn et al., 2009). The rocky reefs examined in this study were selected to establish the baseline of the trophic ecology of two rocky reef areas, with different disturbance levels in the Ecuadorian mainland coast. The presence of *D. mexicanum* was related to the rocky bottoms of LA, where algal presence were more frequent than in the disturbed site (PS). The population density of *E. thouarsii* was higher at the disturbed site (N Cabanillas-Terán, 2013, unpublished data). This study demonstrated that algal abundance is not always equivalent to assimilation by the consumer. For instance, *L. variegata* displayed the lowest dietary contribution at PS and LA for both sea urchins, although it exhibited the highest average biomass at both sites. Grazing preference by *D. mexicanum* and *E. thouarsii* was not related to algal biomass.

The isotopic results characterized different algal assemblages that were specific to each rocky reef bottom (branched macroalgae for LA and turf for PS). The values of *δ*^15^N in algae in this study ranged from 5.05‰ to 9.49‰. This result agreed with the ranges of variation reported in other studies (Owens, 1987). The values of *δ*^13^C fluctuated from −23.65 to −6.90‰ and agreed with data from Fry & Sherr (1984), who reviewed the *δ*^13^C data of benthic algae, noting that values ranged between −30 and −5‰. The different algae species constituting the community of LA showed isotopic values that were closer together, but with a broader cloud distribution in the C vs. N biplot of points relative to what was observed in PS. This suggests a more complex trophic net and shows how primary consumers interact with their resources (McClanahan, 1988; Phillips & Gregg, 2003).

The isotopic ratios of *δ*^15^N could be influenced by two main factors. One factor pertains to changes in dissolved nitrogen, although these changes primarily affect the microscopic algal communities or communities living near upwelling zones (Jennings et al., 1997; Polunin & Pinnegar, 2002). The other factor is the anthropogenic impact (Bode, Alvarez-Ossorio & Varela, 2006), affecting the communities near the coastline. In this case, the community most affected by urban impact was PS, located in front of Manta Port. In this port, human density is higher than 1,000 ind/km^−2^, and artisanal and industrial fishery contribute to nitrogen input, as well as the hotel zone and discharges from tuna processing.

For algae found in both sites (*D. dichotoma*, *L. variegata* and *Polysiphonia* spp.), the average *δ*^15^N were higher in PS. This agrees with other areas with high anthropogenic influence where *δ*^15^N values tended to be higher (Wada, Kadonaga & Matsuo, 1975; Michener & Schell, 1994). Although both localities shared species, the isotopic values for both localities were different because each system had its own structure. The erected branched algae *A. armata* and *Sargassum* spp. (not found in PS), contributed to the structural complexity founded in LA.

Variations in carbon and nitrogen ratios gave us information on trophic spectrum inherent to each site and the contribution of algal species to the sea urchin tissues display information about how consumers assimilate the resources when they inhabit disturbed and/or undisturbed sites. Although both sea urchin species can share the same food resources, we found that their ecological roles were different and there are differences between species in terms of assimilation. This could explain the fact that *δ*^15^N values in the muscle of *E. thouarsii* were higher for both localities, even though both sea urchin species showed a preference for the same species *D. dichotoma*. No overlap of isotope niche breadth of the echinoids was found between the two species (Fig. 3), but the isotopic values between species at PS were closer, suggesting increased competition due to the lack of resources. This result coincided with the mixing model because the two species of sea urchins preferentially consumed similar proportions of the same species. Moreover the SEAc was larger for *E. thouarsii* at both sites, and in LA the niche trophic distance between *D. mexicanum* and *E. thouarsii* was very conspicuous, while in PS the two species of sea urchins are closer (Fig. 3). A low degree of feeding specialization suggests that the sea urchins adapt their foraging behavior to algae availability, being most evident for *E. thouarsii*, that exhibits a broader trophic niche.

The grazing behavior of these sea urchins agreed with the findings by Glynn, Wellington & Birkeland (1979) in the Galápagos Islands, as their grazing was stronger in those areas with 30% or less coral cover. Previous studies highlighted that *E. thouarsii* limited coral growth, as this echinoid interfered with the development of the reef frame and with the ability to modify the habitat structure (Bak, 1994; Carpenter, 1981; Sonnenholzner, Ladah & Lafferty, 2009). We considered *D. mexicanum* to be an important grazer for the rocky bottoms ecosystems, considering that changes in its population caused significant changes in the algal cover of those areas.

Our results supported the evidence that *D. mexicanum* and *E. thouarsii* were coexistent species that play a significant role as herbivores. Nevertheless, they apparently eat whatever they find, and the food items are incorporated differentially between the species. *Diadema mexicanum* grazing effect on algal diversity and community structure is important in the process of formation and maintenance of rocky reefs in Ecuador. This has also been observed in other areas of the ETP where *D. mexicanum* has a relevant role in the recruitment of corals (Alvarado, Cortés & Reyes-Bonilla, 2012). This was also observed in Caribbean reefs with *D. antillarum* (Macintyre, Glynn & Hinds, 2005; Mumby et al., 2006; Idjadi, Haring & Precht, 2010; Sandin & McNamara, 2012), and in shaping the sublittoral ecosystems of the Canary Islands with *D. africanum* (Alves et al., 2003; Tuya et al., 2004; Hernández et al., 2005; Hernández et al., 2008; Sangil et al., 2014; Cabanillas-Terán et al., 2015).

The standard ellipses areas values (Table 5) indicated that niche partitioning may vary depending on different disturbance levels between sites; however, the diets of *D. mexicanum* and *E*. *thouarsii* not only depend on the disturbance condition. For instance, *Dictyota dichotoma* was an important component of the diet of *D. mexicanum* and *E. thouarsii* in the disturbed and undisturbed sites, while *Polysiphonia* spp. was important in disturbed bottoms, where isotopic algal signals are closer to each other. This could lead to a greater number of resource overlap at PS than at LA.

Differential assimilation and niche partitioning are just snapshots. It is important to depict how the shape of the food web varies in time and space (Layman et al., 2007; Schmidt et al., 2007), so it is necessary to carry out more extensive spatial and temporal research. Likewise, it is necessary to deepen research to analyze if the narrower niche amplitude (SEAc) of *D. mexicanum* and its associated presence to scleractian corals (at LA) is consistent to what is happening in the Caribbean, where its presence provides suitable habitat for coral recruitment. The feeding success of herbivores is associated with the competition level for resources; therefore, sympatric species are exposed to a potential trophic overlap. The most pristine zone (LA) exhibited smaller SEAc (considering values per species) and nitrogen values, which indicate a trophic niche partitioning between the main sea urchins on the Ecuadorian coast. However, the limitation of resources could lead to trophic overlap and stronger habitat degradation.

## Supplemental Information

10.7717/peerj.1578/supp-1 Table S1 C:N ratio Mean ±standard deviation values of C:N ratios of algal species and sea urchins taken from of Los Ahorcados (LA) and Perpetuo Socorro (PS).

10.7717/peerj.1578/supp-2 Data S1 Sea urchins density dataset

## Additional Information and Declarations

### Field Study Permissions

The following information was supplied relating to field study approvals (i.e., approving body and any reference numbers): Ministerio de Medio Ambiente de Ecuador 014AT-DPAM-MAE.

### Data Availability

The following information was supplied regarding data availability: Data S1.

## References

[ref-1] Abbott IA, Hollenberg GJ (1976). Marine algae of California.

[ref-2] Afonso-Carrillo J, Sansón M (1999). Algas, hongos y fanerógamas marinas de las islas Canarias.

[ref-3] Alvarado JJ, Cortés J, Reyes-Bonilla H (2012). Bioerosion impact model for the sea urchin *Diadema mexicanum* on three Costa Rican Pacific coral reefs. Revista de Biología Tropical.

[ref-5] Alvarez-Filip L, Dulvy NK, Côté IM, Watkinson AR, Gill JA (2011). Coral identity underpins architectural complexity on Caribbean reefs. Ecological Applications.

[ref-4] Alvarez-Filip L, Dulvy NK, Gill JA, Côté IM, Watkinson AR (2009). Flattening of Caribbean coral reefs: region-wide declines in architectural complexity. Proceedings of the Royal Society of London B.

[ref-6] Alves FMA, Chícharo LM, Serräo E, Abreu AD (2003). Grazing by *Diadema antillarum* (Philippi) upon algal communities on rocky substrates. Scientia Marina.

[ref-7] Andrew NL (1989). Contrasting ecological implications of food limitation in sea urchins and herbivorous gastropods. Marine Ecology Progress Series.

[ref-8] Bak RPM (1994). Sea urchin bioerosion on coral reefs: place in the carbonate budget and relevant variables. Coral Reefs.

[ref-9] Behmer ST, Joern A (2008). Coexisting generalist herbivores occupy unique nutritional feeding niches. Proceedings of the National Academy of Sciences of the United States of America.

[ref-10] Ben-David M, Schell DM (2001). Mixing models in analyses of diet using multiple stable isotopes: a response. Oecologia.

[ref-11] Bode A, Alvarez-Ossorio MT, Varela M (2006). Phytoplankton and macrophyte contributions to littoral food webs in the Galician upwelling estimated from stable isotopes. Marine Ecology Progress Series.

[ref-12] Boecklen WJ, Yarnes CT, Cook BA, James AC (2011). On the use of stable isotopes in trophic ecology. Annual Review in Ecology and Systematics.

[ref-13] Cabanillas-Terán N (2009). Ecología y estatus trófico del erizo de mar *Diadema antillarum* (Philippi, 1845) en los fondos rocosos de las Islas Canarias (Gran Canaria, España). PhD thesis.

[ref-14] Cabanillas-Terán N, Martín JA, Rodríguez-Barreras R, Luque A (2015). Size-density strategy displayed by *Diadema africanum* linked with the stability of urchin-barrens in the Canary Islands. Journal of the Marine Biological Association of the United Kingdom.

[ref-15] Carabel S, Godínez-Dominguez E, Verísimo LF, Freire J (2006). An assessment of sample processing methods for stable isotope analyses of marine food webs. Journal of Experimental Marine Biology and Ecology.

[ref-16] Carpenter RC (1981). Grazing by *Diadema antillarum* (Philippi) and its effects on the benthic algal community. Journal of Marine Research.

[ref-17] Carpenter RC (1986). Partitioning herbivory and its effects on coral reef algal communities. Ecological Monographs.

[ref-18] Cole ML, Valiela I, Kroeger KD, Tomasky GL, Cebrian JC, Wigand J, McKinney RA, Grady SP, Silva MHC (2004). Assessment of a ^15^N isotopic method to indicate anthropogenic eutrophication in aquatic ecosystems. Journal of Environmental Quality.

[ref-19] Cortés J (1997). Biology and geology of coral reefs of the eastern Pacific. Coral Reefs.

[ref-20] Cortés J, Hopley D (2011). Eastern Tropical Pacific coral reefs. The encyclopedia of modern coral reefs: structure, form and process.

[ref-21] Costello MJ, Coll M, Danovaro R, Halpin P, Ojaveer H, Miloslavich P (2010). A census of marine biodiversity knowledge, resources, and future challenges. PLoS ONE.

[ref-22] DeNiro MJ, Epstein S (1981). Influence of diet on the distribution of nitrogen isotopes in animals. Geochimica et Cosmochimica Acta.

[ref-23] Duffy JE, Cardinale BJ, France KE, McIntyre PB, Thébault E, Loreau M (2007). The functional role of biodiversity in ecosystems: incorporating trophic complexity. Ecology Letters.

[ref-24] Dumas P, Kulbicki M, Chifflet S, Fichez R, Ferraris J (2007). Environmental factors influencing urchin spatial distributions on disturbed coral reefs (New Caledonia, South Pacific). Journal of Experimental Marine Biology and Ecology.

[ref-26] Fry B (2006). Stable isotope ecology.

[ref-25] Fry B, Sherr E (1984). *δ*^13^C measurements as indicators of carbon flow in marine and freshwater ecosystems. Contribution to Marine Science.

[ref-27] Glynn PW (1993). Coral reef bleaching: ecological perspectives. Coral Reefs.

[ref-28] Glynn PW, Cortés J (2003). Coral communities and coral reefs of Ecuador. Latin American coral reefs.

[ref-29] Glynn PW (2004). High complexity food webs in low-diversity Eastern Pacific reef–coral communities. Ecosystems.

[ref-32] Glynn PW, Maté JL, Baker AC, Calderón MO (2001). Coral bleaching and mortality in panama and Ecuador during the 1997–1998 El Niño–Southern Oscillation Event: spatial/temporal patterns and comparisons with the 1982–1983 event. Bulletin of Marine Science.

[ref-33] Glynn PW, Riegl B, Romanski AMS, Baums IB (2009). Rapid recovery of a coral reef at Darwin Island, Galapagos Islands. Galapagos Research.

[ref-30] Glynn PW, Wellington GM (1983). Corals and coral reefs of the Galápagos Islands.

[ref-31] Glynn PW, Wellington GM, Birkeland C (1979). Coral reef growth in the Galápagos: limitation by sea urchins. Science.

[ref-34] Graham NAJ, Nash KL (2013). The importance of structural complexity in coral reef ecosystems. Coral Reefs.

[ref-35] Guidetti P (2006). Marine reserves reestablish lost predatory interactions and cause community effects in rocky reefs. Ecological Applications.

[ref-36] Guzmán HM, Cortés J (1993). Los arrecifes coralinos del Pacífico Oriental Ecuatorial: revisión y perspectivas. Revista de Biología Tropical.

[ref-37] Hamaoka H, Okuda N, Fukumoto T, Miyasaka H, Omori K, Ohkouchi N, Tayasu I, Koba K (2010). Seasonal dynamics of a coastal food web: stable isotope analysis of a higher consumer. Earth, life, and isotopes.

[ref-38] Hay ME (1984). Predictable spatial escapes from herbivory: how do these affect the evolution of herbivore resistance in tropical marine communities?. Oecologia.

[ref-39] Hay ME, Fenical W (1988). Marine plant-herbivore interactions: the ecology of chemical defense. Annual Review of Ecology and Systematic.

[ref-40] Hereu B, Garcia-Rubies A, Linares C, Navarro L, Bonaviri C, Cebrian E, Zabala M (2012). Impact of the Sant Esteve’s storm (2008) on the algal cover in infralittoral rocky photophilic communities. Final report.

[ref-41] Hernández JC, Clemente S, Brito A, Falcon JM, García N, Barquin J (2005). Estado de las poblaciones de *Diadema antillarum* (Echinoidea: Diadematidae) y del recubrimiento de macroalgas en las Reservas Marinas de Canarias: patrones de distribución espacial. Vieraea.

[ref-42] Hernández JC, Clemente S, Sangil C, Brito A (2008). The key role of the sea urchin *Diadema* aff. *antillarum* in controlling macroalgae assemblages throughout the Canary Island (eastern subtropical Atlantic): an spatio-temporal approach. Marine Environmental Research.

[ref-44] Hoegh-Guldberg O (1999). Climate change, coral bleaching and the future of the world’s coral reefs. Marine and Freshwater Research.

[ref-45] Hunter MD, Price PW (1992). Playing chutes and ladders: heterogeneity and the relative roles of bottom-up and top-down forces in natural communities. Ecology.

[ref-46] Idjadi JA, Haring RN, Precht WF (2010). Recovery of the sea urchin *Diadema antillarum* promotes scleractinian coral growth and survivorship on shallow Jamaican reefs. Marine Ecology Progress Series.

[ref-47] Jackson AL, Inger R, Parnell AC, Bearhop S (2011). Comparing isotopic niche widths among and within communities: SIBER–Stable Isotope Bayesian Ellipses in R. Journal of Animal Ecology.

[ref-48] Jennings S, Reñones O, Morales-Nin B, Polunin NVC, Moranta J, Coll J (1997). Spatial variation in the ^15^N and ^13^C stable isotope composition of plants, invertebrates and fishes on Mediterranean reefs: implications for the study of trophic pathways. Marine Ecology Progress Series.

[ref-49] Karube Z, Sakai Y, Takeyama T, Okuda N, Kohzu A, Yoshimizu C, Nagata T, Tayasu I (2010). Carbon and nitrogen stable isotope ratios of macroinvertebrates in the littoral zone of Lake Biwa as indicators of anthropogenic activities in the watershed. Ecological Research.

[ref-51] Lapointe BE, Barile PJ, Littler MM, Littler DS (2005). Macroalgal blooms on southeast Florida coral reefs: II. Cross-shelf discrimination of nitrogen sources indicates widespread assimilation of sewage nitrogen. Harmful Algae.

[ref-52] Lapointe BE, Langton R, Bedford BJ, Potts AC, Day O, Hu C (2010). Land-based nutrient enrichment of the Buccoo Reef Complex and fringing coral reefs of Tobago, West Indies. Marine Pollution Bulletin.

[ref-54] Lawrence JM (1975). On the relationships between marine plants and sea urchins. Oceanography and Marine Biology, An Annual Review.

[ref-53] Layman CA, Quattrochi JP, Peyer CM, Allgeier JE (2007). Niche width collapse in a resilient top predator following ecosystem fragmentation. Ecology Letters.

[ref-55] Littler DS, Littler MM (2010). Marine plants of Pacific Panama.

[ref-56] Macintyre IG, Glynn PW, Hinds F (2005). Evidence of the role of *Diadema antillarum* in the promotion of coral settlement and survivorship. Coral Reefs.

[ref-57] Martínez-Ortiz J, Galván-Magaña F, Carrera-Fernández M, Mendoza-Intriago D, Estupiñán-Montaño C, Cedeño-Figueroa L, Martínez-Ortíz J, Galván-Magaña F (2007). Abundancia estacional de tiburones desembarcados en Manta-Ecuador/Seasonal abundante of Sharks landings in Manta-Ecuador. Tiburones en el Ecuador: Casos de estudio.

[ref-58] McClanahan TR (1988). Coexistence in a sea urchin guild and its implications to coral reef diversity and degradation. Oecologia.

[ref-59] Michener RH, Schell DM, Lajtha K, Michener R (1994). Stable isotope ratios as tracers in marine aquatic foodwebs. Stable isotopes in ecology and environmental science.

[ref-60] Minagawa M, Wada E (1984). Stepwise enrichment of ^15^N along food chains: further evidence and the relation between ^15^N and animal age. Geochimica et Cosmochimica Acta.

[ref-61] Moore JW, Semmens BX (2008). Incorporating uncertainty and prior information into stable isotope mixing models. Ecology Letters.

[ref-62] Mumby PJ, Foster NL, Fahy EAG (2005). Patch dynamics of coral reef macroalgae under chronic and acute disturbance. Coral Reefs.

[ref-63] Mumby PJ, Hedley JD, Zychaluk K, Harborne AR, Blackwell PG (2006). Revisiting the catastrophic die-off of the urchin *Diadema antillarum* on Caribbean coral reefs: fresh insights on resilience from a simulation model. Ecological Modelling.

[ref-64] Newsome SD, Martinez del Rio C, Bearhop S, Phillips DL (2007). A niche for isotopic ecology. Frontiers in Ecology and the Environment.

[ref-65] Owens NJP (1987). Natural variation in ^15^N in the marine environment. Advances in Marine Biology.

[ref-66] Parnell AC, Inger R, Bearhop S, Jackson AL (2010). Source partitioning using stable isotopes: coping with too much variation. PLoS ONE.

[ref-67] Parnell AC, Jackson AL (2013). SIAR: stable isotope analysis in R.

[ref-70] Peterson BJ (1999). Stable isotopes as tracers of organic matter input and transfer in benthic food webs: a review. Acta Oecologica.

[ref-69] Peterson BJ, Fry B (1987). Stable isotopes in ecosystem studies. Annual Review of Ecology, Evolution, and Systematics.

[ref-68] Peterson BJ, Howarth RW, Garrett RH (1985). Multiple stable isotopes used to trace the flow of organic matter flow in estuarine food webs. Science.

[ref-71] Phillips DL (2012). Converting isotope values to diet composition: the use of mixing models. Journal of Mammalogy.

[ref-72] Phillips DL, Gregg JW (2003). Source partitioning using stable isotopes: coping with too many sources. Oecologia.

[ref-75] Phillips DL, Inger R, Bearhop S, Jackson AL, Moore JW, Parnell AC, Semmens BX, Ward EJ (2014). Best practices for use of stable isotope mixing models in food web studies. Canadian Journal of Zoology.

[ref-73] Phillips DL, Koch PL (2002). Incorporating concentration dependence in stable isotope mixing models. Oecologia.

[ref-74] Phillips NE, Shima JS (2006). Differential effects of suspended sediments on larval survival and settlement of New Zealand urchins *Evechinus chloroticus* and abalone *Haliotis iris*. Marine Ecology Progress Series.

[ref-77] Polunin NVC, Pinnegar JK, Hart PJB, Reynolds JD (2002). Trophic ecology and the structure of marine food webs. Handbook of fish biology and fisheries, volume 1: fish biology.

[ref-76] Polunin NV, Polunin C, Morales-Nin B, Pawsey WE, Carles JE, Pinnegar JK, Moranta J (2001). Feeding relationships in Mediterranean bathyal assemblages elucidated by stable nitrogen and carbon isotope data. Marine Ecology Progress Series.

[ref-78] Post DM (2002). Using stable isotopes to estimate trophic position models, methods, and assumptions. Ecology.

[ref-79] Post DM, Layman CA, Arrington DA, Takimoto G, Quattrochi J, Montaña CG (2007). Getting to the fat of the matter: models, methods and assumptions for dealing with lipids in stable isotope analyses. Oecologia.

[ref-80] R Core Team (2014). R: a language and environment for statistical computing.

[ref-81] Reaka-Kudla ML, Feingold JS, Glynn PW (1996). Experimental studies of rapid bioerosion of coral reefs in the Galapagos Islands. Coral Reefs.

[ref-82] Risk MJ (1972). Fish diversity on a coral reef in the Virgin Islands. Atoll Research Bulletin.

[ref-83] Rivera F, Martínez PC (2011). *Guía fotográfica de corales y octocorales: Parque Nacional Machalilla y Reserva de Producción Faunística Marino Costera Puntilla de Santa Elena*. Con contribuciones de Odalisca Breedy.

[ref-84] Rodríguez SR (2003). Consumption of drift kelp by intertidal populations of the sea urchin *Tetrapygus niger* on the central Chilean coast: possible consequences at different ecological levels. Marine Ecology Progress Series.

[ref-85] Rodríguez-Barreras R, Cuevas E, Cabanillas-Terán N, Sabat AM (2015). Potential omnivory in the sea urchin *Diadema antillarum*?. Regional Studies in Marine Science.

[ref-86] Rossi S (2013). The destruction of the ‘animal forests’ in the oceans: towards an over-simplification of the benthic ecosystems. Ocean and Coastal Management.

[ref-87] Sala E, Boudouresque CF, Harmelin-Vivien M (1998). Fishing, trophic cascades and the structure of algal assemblages: evaluation of an old but untested paradigm. Oikos.

[ref-88] Sandin SA, McNamara DE (2012). Spatial dynamics of benthic competition on coral reefs. Oecologia.

[ref-89] Sangil C, Sansón M, Díaz-Villa T, Hernández JC, Clemente S, Afonso-Carrillo J (2014). Spatial variability, structure and composition of crustose algal communities in *Diadema africanum* barrens. Helgoland Marine Research.

[ref-90] Schmidt SN, Olden JD, Solomon CT, Vander-Zanden MJ (2007). Quantitative approaches to the analysis of stable isotope food web data. Ecology.

[ref-91] Sonnenholzner JI, Ladah LB, Lafferty KD (2009). Cascading effects of fishing on Galapagos rocky reef communities: reanalysis using corrected data. Marine Ecology Progress Series.

[ref-92] Steneck RS, Reaka ML (1983). Quantifying herbivory on coral reefs: just scratching the surface and still biting off more than we can chew. The ecology of deep and shallow coral reefs, symposia series for undersea research.

[ref-93] Steneck RS, Dethier MN (1994). A functional group approach to the structure of algal-dominated communities. Oikos.

[ref-94] Tomas F, Álvarez-Cascos D, Turon X, Romero J (2006). Differential element assimilation by sea grass urchin *Paracentrotus lividus* in seagrass beds: implications for trophic interactions. Marine Ecology Progress Series.

[ref-95] Tuya F, Boyra A, Sanchez-Jerez P, Barbera C, Haroun RJ (2004). Relationships between rocky-reef fish assemblages, the sea urchin *Diadema antillarum* and macroalgae throughout the Canarian Archipelago. Marine Ecology Progress Series.

[ref-96] Underwood AJ, John DM, Hawkins SJ, Price JH (1992). Competition and marine plant-animal interactions. Plant-animal interactions in the marine benthos.

[ref-99] Vanderklift MA, Kendrick GA, Smith AJ (2006). Differences in trophic position among sympatric sea urchin species. Estuarine, Coastal and Shelf Science.

[ref-97] Vander-Zanden MJ, Rasmussen JB (1996). A trophic position model of pelagic food webs: impact on contaminant bioaccumulation in lake trout. Ecological Monographs.

[ref-98] Vander-Zanden MJ, Rasmussen JB (2001). Variation in *δ*^15^N and *δ*^13^C trophic fractionation: implications for aquatic food web studies. Limnology and Oceanography.

[ref-100] Villón C, Beltrán X (1998a). Diagnóstico de la actividad pesquera en el puerto de Manta, Provincia de Manabí. *Boletín Científico y Técnico*.

[ref-101] Villón C, Beltrán X (1998b). Diagnóstico de la actividad pesquera en el puerto de San Mateo, provincia de Manabí. Boletín Científico y Técnico.

[ref-102] Wada E, Kadonaga T, Matsuo S (1975). ^15^N abundance in nitrogen of naturally occurring substances and global assessment of denitrification from isotopic viewpoint. Geochemical Journal.

[ref-103] Wilkinson C (1999). Global and local threats to coral reef functioning and existence: review and predictions. Marine and Freshwater Research.

[ref-104] Wing SR, McLeod RJ, Clark KL, Frew RD (2008). Plasticity in the diet of two echinoderm species across an ecotone: microbial recycling of forest litter and bottom-up forcing of populations structure. Marine Pollution Bulletin.

[ref-105] Zar JH (2010). Biostatistical analysis.

