# Supplementary material for: Trophic ecology of sea urchins in coral-rocky reef systems, Ecuador
Source: PeerJ. 2016 Jan 14;4:e1578. doi: 10.7717/peerj.1578 (PMC4734443; doi:10.7717/peerj.1578)
Supplement: Table S1 — Mean ±standard deviation values of C:N ratios of algal species and sea urchins taken from of Los Ahorcados (LA) and Perpetuo Socorro (PS). [file peerj-04-1578-s001.docx]

**Table S1.** Mean ± standard deviation values of C:N ratios of algal species and sea urchins taken from of Los Ahorcados (LA) and Perpetuo Socorro (PS).

| **Algal species** | **LA** | |  | **PS** | |  |
| --- | --- | --- | --- | --- | --- | --- |
|  | **C:N** | |  | **C:N** | |  |
| *A. armata* (n=4) | 8.48 | ±0.19 | - | - |  | |
| *D. dichotoma* (n=4) | 13.59 | ±5.31 | *D. dichotoma* (n=3) | 13.74 | ±3.15 | |
| *L. variegata* (n=4) | 17.91 | ±3.68 | *L. variegata* (n=3) | 20.03 | ±0.47 | |
| *Polysiphonia* spp*.*  (n=6) | 15.89 | ±2.80 | *Polysiphonia* spp. (n=4) | 13.23 | ±1.99 | |
| *Sargassum* spp. (n=4) | 16.38 | ±1.70 | - | - |  | |
| **Sea urchin species** |  |  |  |  |  | |
| *D. mexicanum* (n=4) | 3.50 | ±0.05 | *D. mexicanum* (n=12) | 3.48 | ±0.34 | |
| *E. thouarsii* (n=6) | 3.48 | ±0.15 | *E. thouarsii* (n=8) | 3.47 | ±0.22 | |
